# Real‐World Determinants of Treatment Selection in Frontline Chronic Lymphocytic Leukemia: The FIRST‐CLL Study

**DOI:** 10.1002/hon.70246

**Published:** 2026-09-16

**Authors:** Francesca Perutelli, Matteo Arata, Raffaella Pasquale, Sara Pepe, Maura Nicolosi, Marina Deodato, Ilaria Piras, Erika Fioribello, Alessandro Bosi, Riccardo Moia, Andrea Ferrario, Michela Schirru, Elia Boccellato, Martina Bullo, Daniela Gottardi, Myriam Foglietta, Andrea Galitzia, Marta Coscia, Gianluca Gaidano, Michele Merli, Chiara Salvetti, Roberta Murru, Anna Maria Frustaci, Roberto Freilone, Jacopo Olivieri, Francesca Romana Mauro, Benedetto Bruno, Candida Vitale

**Affiliations:** ^1^ Department of Molecular Biotechnology and Health Sciences University of Torino & University Division of Hematology A.O.U. Città della Salute e della Scienza di Torino Torino Italy; ^2^ SOC Clinica Ematologica—Centro Trapianti e Terapie Cellulari Azienda Sanitaria Universitaria Friuli Centrale (ASUFC) Udine Italy; ^3^ Hematology Department of Translational and Precision Medicine Sapienza University of Roma Roma Italy; ^4^ Division of Hematology A.O.U. Città della Salute e della Scienza di Torino Torino Italy; ^5^ Department of Haematology Niguarda Cancer Center ASST Grande Ospedale Metropolitano Niguarda Milano Italy; ^6^ Hematology and Stem Cell Transplantation Unit Ospedale Oncologico A. Businco ARNAS G. Brotzu Cagliari Italy; ^7^ S.S.D. Microcitemia, anemie congenite e dismetabolismo del ferro E.O. Ospedali Galliera & Clinic of Hematology Department of Internal Medicine and Medical Specialties University of Genova Genova Italy; ^8^ Division of Hematology Fondazione IRCCS Ca’ Granda Ospedale Maggiore Policlinico Milano Italy; ^9^ Division of Hematology Department of Translational Medicine University of Eastern Piedmont Novara Italy; ^10^ UOC Ematologia Azienda Socio Sanitaria Territoriale dei Sette Laghi Ospedale di Circolo di Varese Varese Italy; ^11^ Struttura Complessa di Ematologia Ospedale San Francesco ASL Nuoro Nuoro Italy; ^12^ Division of Hematology Azienda Santa Croce e Carle Cuneo Italy; ^13^ Division of Hematology Mauriziano Hospital Torino Italy; ^14^ Department of Medicine and Surgery University of Insubria & UOC Ematologia Azienda Socio Sanitaria Territoriale dei Sette Laghi Ospedale di Circolo di Varese Varese Italy; ^15^ Clinic of Hematology IRCCS Ospedale Policlinico San Martino Genova Italy

## Abstract

Multiple targeted agents are available for first‐line treatment of chronic lymphocytic leukemia (CLL), yet the absence of mature head‐to‐head comparisons makes treatment selection challenging. The prospective, multicenter FIRST‐CLL study evaluated real‐world treatment allocation, and the contribution of biological, clinical, logistical and patient‐related factors to first‐line regimen choice in Italy. 285 consecutive patients with CLL/small lymphocytic lymphoma initiating first‐line therapy across 13 Centers were included. Treating physicians rated predefined variables guiding treatment selection using the 5‐point *relevance score*. Overall, 116 patients (41%) received continuous Bruton tyrosine kinase inhibitors (BTKi), 114 (40%) ibrutinib plus venetoclax (IV), and 55 (19%) venetoclax plus obinutuzumab (VO). Continuous BTKi were more frequently prescribed in older and comorbid patients, whereas IV was preferentially used in younger individuals. *TP53* disruption was associated with BTKi use, while mutated immunoglobulin heavy chain status was common in patients receiving VO. According to *relevance scores*, age, treatment duration and administration route, and patient preference were the most influential determinants of treatment selection. Comorbidity burden additionally guided BTKi prescription, whereas molecular features were more relevant in the selection of fixed‐duration regimens. This study offers a real‐world quantitative assessment of treatment decision drivers in frontline CLL, offering a data‐driven framework to interpret clinical decision‐making.

## 1 Introduction

Treatment options for patients with chronic lymphocytic leukemia (CLL) have rapidly increased in recent years, with multiple oral, chemoimmunotherapy‐free agents now available from the first line of treatment [1, 2, 3, 4, 5, 6, 7]. Bruton tyrosine kinase inhibitors (BTKi)—including ibrutinib and the next‐generation agents acalabrutinib and zanubrutinib—are administered continuously until progression or intolerance, while the anti‐Bcl2 compound venetoclax—combined with the anti‐CD20 monoclonal antibody obinutuzumab—offers a fixed‐duration alternative of one year. More recently, the combination of ibrutinib plus venetoclax (IV) has been approved by the European Medicines Agency as a further fixed‐duration option for treatment‐naïve patients with CLL.

While this therapeutic progress has dramatically improved patient outcomes, it has also introduced complexity in treatment selection, as clinicians must choose among several highly effective options in the absence of mature superiority comparative data. Selecting the optimal first‐line regimen requires balancing multiple factors, including CLL biological risk features, regimen‐specific toxicities, patients' fitness and comorbidities, logistical consideration, and patient preference. While some of these aspects are well‐established, others are more subjective and may vary in importance among clinicians and clinical contexts.

Biological risk stratification plays a central role in therapeutic decision‐making, as reflected in current guideline recommendations [1]. *TP53* aberrations and immunoglobulin heavy chain variable region (IGHV) status are recognized prognostic factors and may guide the choice between continuous and fixed‐duration strategies.

Treatment‐related toxicities also influence therapeutic decisions, particularly cardiovascular toxicity for BTKi and tumor lysis syndrome (TLS) risk for venetoclax‐based regimens [8, 9, 10, 11]. Notably, in an older population frequently characterized by comorbidities and polypharmacy, the balance between efficacy and safety becomes particularly relevant.

Continuous BTKi and fixed‐duration venetoclax‐based regimens differ substantially also in administration schedules and monitoring requirements, introducing additional logistical and patient‐centered considerations.

Despite this complex interplay, limited real‐world evidence exists on how clinicians prioritize these factors when selecting first‐line therapy for CLL, and how these priorities differ across currently available regimens.

Based on this background, the present study aims at describing real‐world allocation of first‐line therapies in CLL across multiple Italian Centers and at quantifying the relative importance of clinical, biological, logistical and patient‐related parameters in physicians' treatment decisions.

## 2 Methods

This is a prospective multicenter observational study conducted in Italy. Data were collected from consecutive CLL/small lymphocytic lymphoma (SLL) patients with an indication to start a first‐line therapy according to the IWCLL 2018 criteria [12]. Enrolled patients started treatment from February 2024 to July 2025, outside of clinical trials, and received a chemotherapy‐free regimen approved and reimbursed by the Italian National Health System. Treatment decisions were determined by the treating physicians as part of routine clinical care, and were not driven by study participation.

Baseline demographic, clinical and prognostic characteristics were recorded through chart review. For each patient, treating physicians rated on a five‐point scale (*relevance score*, 1 = not relevant to 5 = very relevant) the perceived importance of predefined parameters in guiding treatment selection, including prognostic features, TLS risk, lymph nodes size, patient's age and comorbidities, concomitant treatments, route and timing of administration, patient's preference and availability of the regimen at the Center.

The study was conducted in accordance with the Declaration of Helsinki and was approved by the appropriate institutional ethics committees of all participating Centers. Written informed consent was obtained from patients prior to data collection.

All statistical analyses were performed using *IBM SPSS Statistics*, version 29.0.1.0 (IBM Corp., Armonk, NY, USA).

Continuous variables were assessed in terms of distribution using the Shapiro–Wilk test. When data were normally distributed, group comparisons were performed using one‐way analysis of variance (ANOVA); for non‐normal distributions, the Kruskal–Wallis test was applied, followed by Dunn's post hoc test with Bonferroni adjustment when appropriate. For two‐group comparisons, the Student's t‐test or Mann–Whitney *U* test were used as appropriate.

Categorical variables were described as counts and percentages, and comparisons among treatment groups were carried out using the Pearson's Chi‐square test or Fisher's exact test when > 20% of expected cell counts were below 5 or any expected count was < 1. The Fisher–Freeman–Halton exact test was used for categorical variables with more than two levels and small sample sizes.

All tests were two‐sided, and a *p*‐value < 0.05 was considered statistically significant. *p*‐values lower than 0.001 are reported as *p* < 0.001.

## 3 Results

Data from 285 patients with CLL/SLL starting first‐line therapy at 13 Italian Centers were collected. Overall, 116 patients (41%) received a continuous BTKi (zanubrutinib, *n* = 56; acalabrutinib, *n* = 59; ibrutinib *n* = 1), 114 (40%) ibrutinib plus venetoclax (IV) and 55 (19%) venetoclax plus Obinutuzumab (VO).

Baseline characteristics of patients included in the study are reported in Table 1.

**TABLE 1:** Characteristics of the whole cohort of patients enrolled in the study, and of each treatment group.

	Whole cohort (*n* = 285)	BTKi (*n* = 116)	IV (*n* = 114)	VO (*n* = 55)
Age, median (range)	72 (34–91) [*n* = 285]	**79 (52‐91) [*n* = 116]**	**66 (36‐82) [*n* = 114]**	**70 (34‐86) [*n* = 55]** ^a^
Males, number (%)	167 (59) [*n* = 285]	**58 (50) [*n* = 116]**	**66 (58) [*n* = 114]**	**43 (78) [*n* = 55]** ^b^
Lymphocyte count, × 10^9^/L, median (range)	67.8 (0.7–528) [*n* = 279]	71.1 (1–528) [*n* = 116]	73.6 (1.3–382) [*n* = 114]	53.8 (0.7–326) [*n* = 55]
Hemoglobin, g/dL, median (range)	11.3 (6.4–16) [*n* = 285]	10.3 (5.1–15) [*n* = 116]	10.8 (5.2–16) [*n* = 114]	11.6 (7–16.2) [*n* = 55]
Platelet count, × 10^9^/L, median (range)	130 (9–389) [*n* = 285]	129 (11–389) [*n* = 116]	137 (9–379) [*n* = 114]	122 (38–219) [*n* = 55]
RAI stage, number (%)	[*n* = 285]	[*n* = 116]	[*n* = 114]	[*n* = 55]
I	24 (8)	9 (7)	10 (9)	5 (9)
II	85 (30)	28 (24)	38 (33)	19 (35)
III	89 (31)	43 (37)	37 (32)	9 (16)
IV	86 (31)	36 (31)	28 (26)	22 (40)
Binet stage, number (%)	[*n* = 285]	[*n* = 116]	[*n* = 114]	[*n* = 55]
A	16 (6)	6 (5)	5 (4)	5 (9)
B	106 (37)	33 (28)	51 (45)	22 (40)
C	163 (57)	77 (66)	58 (51)	28 (51)
CIRS score, median (range)	4 (0–15) [*n* = 285]	**5 (0‐14) [*n* = 116]**	**3 (0‐15) [*n* = 114]** ^c^	4 (0–13) [*n* = 55]
Elevated LDH, number (%)	137 (48) [*n* = 285]	61 (53) [*n* = 116]	54 (47) [*n* = 114]	22 (40) [*n* = 55]
Beta2‐microglobulin, mg/L, median (range)	5 (1.4–19.4) [*n* = 256]	4.3 (1.9–19.4) [*n* = 99]	3.8 (1.4–16.3) [*n* = 107]	3.7 (1.4–16.7) [*n* = 50]
Severe hypogammaglobulinemia (IgG < 300 mg/dL), number (%)	124 (44) [*n* = 283]	53 (46) [*n* = 114]	45 (39) [*n* = 114]	26 (47) [*n* = 54]
IGHV unmutated, number (%)	151 (55) [*n* = 272]	**77 (72) [*n* = 107]**	**60 (55) [*n* = 110]**	**14 (25) [*n* = 55]** ^d^
FISH abnormalities, number (%)^e^	[*n* = 268]	[*n* = 104]	[*n* = 110]	[*n* = 54]
Deletion 13q	80 (30)	35 (34)	29 (26)	23 (43)
Negative	99 (37)	28 (27)	44 (40)	20 (37)
Trisomy 12	47 (18)	16 (15)	21 (19)	10 (19)
Deletion 11q	27 (10)	11 (11)	15 (13)	1 (2)
Deletion 17p	13 (5)	**13 (13)**	**0**	**0** ^**^**^
*TP53* mutated, number (%)	20 (7) [*n* = 268]	**17 (15) [*n* = 115]**	**3 (3) [*n* = 110]**	**0 [*n* = 55]** ^d^
*TP53* disruption [i.e. del(17p) and/or *TP53* mutation]	24 (9) [*n* = 268]	**21 (20) [*n* = 104]**	**3 (3) [*n* = 110]**	**0 [*n* = 55]** ^d^
Patients with lymph nodes ≥ 5 cm, number (%)	125 (44) [*n* = 285]	48 (41) [*n* = 116]	59 (52) [*n* = 114]	18 (33) [*n* = 55]
TLS risk, number (%)	[*n* = 285]	[*n* = 116]	[*n* = 114]	[*n* = 55]
Low	29 (11)	16 (14)	8 (7)	5 (9)
Intermediate	155 (54)	61 (53)	59 (52)	35 (64)
High	101 (35)	39 (34)	47 (41)	15 (27)
Work status, number (%)	[*n* = 285]	**[*n* = 116]**	**[*n* = 114]**	**[*n* = 55]**
Retired/unemployed	182 (64)	**105 (90)**	**46 (40)**	**31 (56)**
Active worker	103 (36)	**11 (10)**	**68 (60)**	**24 (44)** ^d^
Availability of caregivers, number (%)	[*n* = 279]	[*n* = 111]	[*n* = 114]	[*n* = 54]
Very supportive caregiver	56 (20)	26 (23)	20 (17)	10 (17)
Caregiver present	168 (60)	65 (59)	66 (58)	37 (69)
No caregiver/minor support	55 (20)	20 (18)	28 (25)	7 (13)

*Note:* Bold values are statistically significant results.

Abbreviations: CIRS, cumulative illness rating scale; FISH, fluorescent in situ hybridization; IGHV, immunoglobulin heavy chain variable region; TLS, tumor lysis syndrome.

^a^ *p* = 0.031 when comparing IV versus VO groups; *p* = 0.001 when comparing BTKi versus IV groups; *p* = 0.001 when comparing VO versus BTKi groups.

^b^ *p* = 0.02 for all comparisons.

^c^ *p* < 0.001 only when comparing BTKi versus IV groups.

^d^ *p* < 0.001 for all comparisons.

^e^ Grouped according to Dohner's hierarchical classification.

### 3.1 Age

**FIGURE 1:**
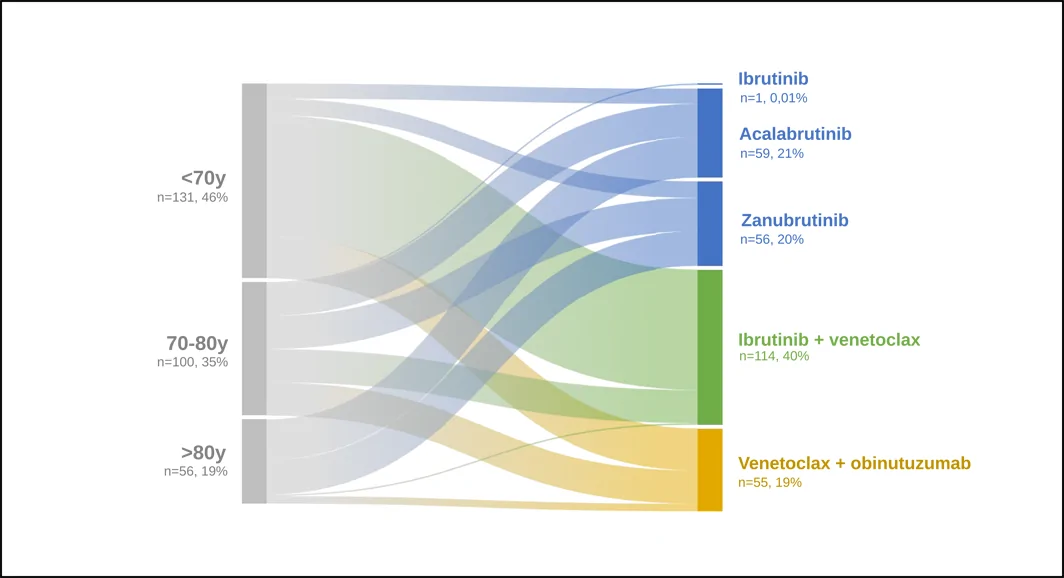
Allocation of first‐line treatments according to patients' age.

Patients receiving continuous BTKi were significantly older than those treated with a fixed‐duration regimen (median age: 79 for BTKi vs. 66 for IV and 70 for VO; *p* < 0.001 for both comparisons), with a significant age difference observed also between the IV and VO groups (*p* = 0.031). Accordingly, IV was the most prescribed regimen in patients < 70 years old (80/131, 62%), whereas in patients > 80 years old, continuous BTKi were largely preferred (50/56, 89%; acalabrutinib, *n* = 27; zanubrutinib, *n* = 23). Figure 1 depicts the allocation of first‐line treatments according to patients' age.

### 3.2 Molecular Prognostic Features

**FIGURE 2:**
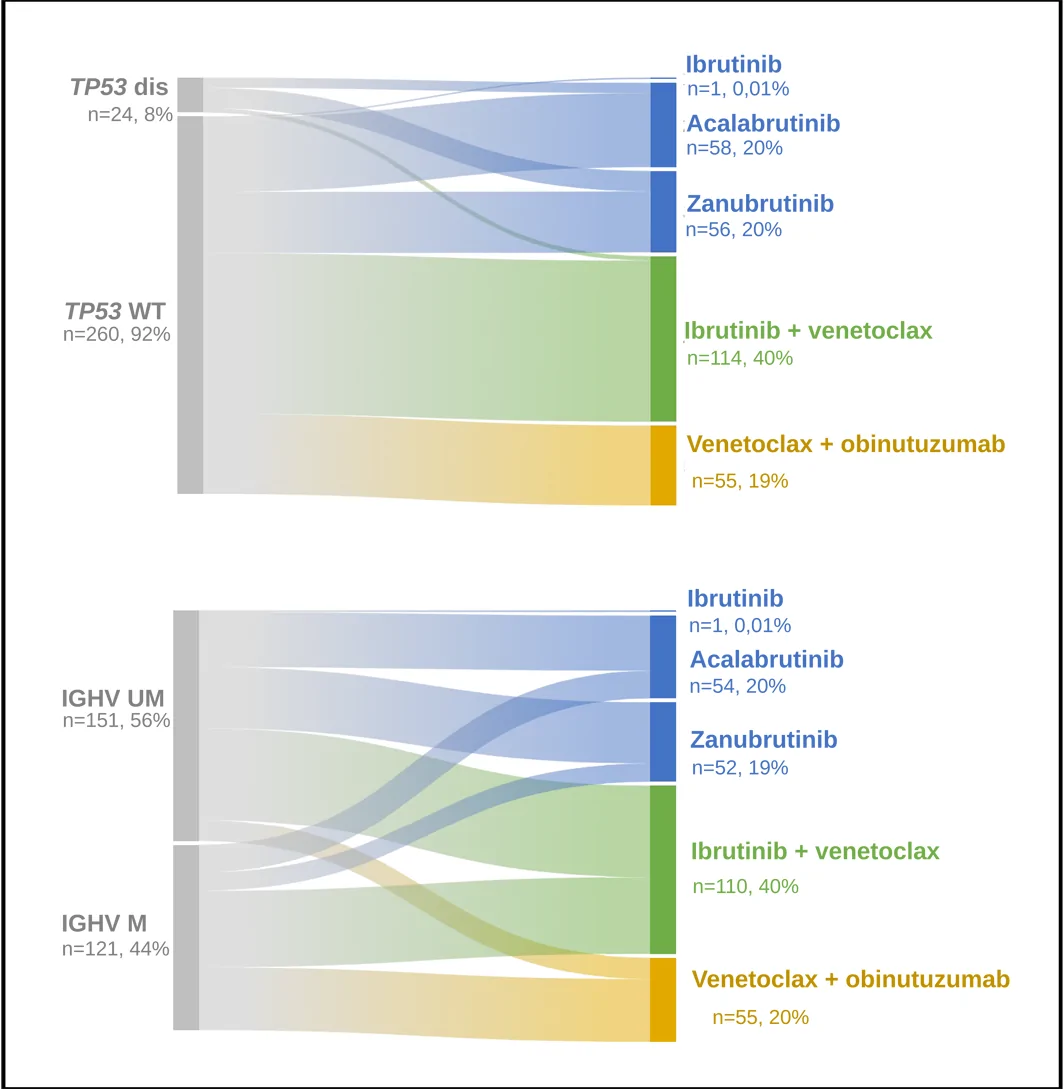
Allocation of first‐line treatments according to molecular prognostic features. Dis, disrupted; IGHV, immunoglobulin heavy chain variable region; M, mutated; UM, unmutated; WT, wild type. For *TP53* evaluation, data available for 284 patients. For IGHV status, data available for 272 patients.

Prognostic factors were evaluated across treatment groups (Table 1). The distribution of *TP53* disruption [i.e., del(17p) and/or *TP53* mutation] and IGHV mutational status differed significantly among patients treated with BTKi, IV or VO (*p* < 0.001). The BTKi cohort was enriched for patients with a *TP53* disruption, and in the VO cohort the majority of patients (75%) carried a mutated IGHV status. Among patients with *TP53* disruption (*n* = 24), the majority received zanubrutinib (*n* = 14, 58%), 7 (29%) were treated with acalabrutinib and three patients (13%) received the IV regimen. Patients carrying unmutated IGHV status (*n* = 151) were preferentially treated with a BTKi‐containing regimen (continuous BTKi, *n* = 77, 51%; IV, *n* = 60, 40%; VO, *n* = 14, 9%). Figure 2 illustrates the distribution of first‐line treatments by molecular features.

### 3.3 Lymph Node Size and TLS Risk

In the overall cohort, 44% of patients presented with lymph nodes ≥ 5 cm, and TLS risk was intermediate or high in the vast majority of cases (89%) (Table 1). When examining the distribution of these features across treatment groups, no significant differences were observed.

### 3.4 Comorbidities and Concomitant Therapies

Patients' fitness was assessed through the Cumulative Illness Rating Scale (CIRS) score. Results showed a significantly higher comorbidity burden in BTKi‐treated patients compared with the IV group (*p* < 0.001), whereas no significant differences were observed between IV and VO or between VO and BTKi (median CIRS score: overall 4; BTKi, 5; IV, 3; VO, 4) (Table 1).

The association between baseline comorbidities of relevance and treatment allocation was evaluated by comparing patients with and without each condition (Table 2). Hypertensive patients (*n* = 130) were more frequently treated with second‐generation BTKi (54%), whereas IV (33%) and VO (12%) were less commonly prescribed in this subgroup (*p* < 0.001). A similar pattern was observed for patients with a history of cardiac arrhythmias (*n* = 33; *p* = 0.002), with affected individuals more often receiving second‐generation BTKi (54%) or VO (33%), and rarely IV (12%). Although based on a limited number of cases (*n* = 11), heart failure was significantly associated with treatment choice (*p* = 0.013), with most affected patients receiving acalabrutinib (55%), followed by zanubrutinib (27%). Patients with chronic kidney disease (CKD, *n* = 42) were more frequently treated with continuous BTKi (62%) and less frequently with IV (19%) or VO (19%) (*p* = 0.008).

**TABLE 2:** Allocation of different treatments based on the presence of specific comorbidities at baseline.

	Hypertension (*n* = 130)	History of cardiac arrhythmias (*n* = 33)	Heart failure (*n* = 11)	Recent major infection (*n* = 13)	Chronic kidney disease (*n* = 42)	Recent major bleeding (*n* = 4)
Acalabrutinib	38 (29%)	7 (21%)	6 (55%)	3 (23%)	15 (36%)	0
Zanubrutinib	33 (25%)	11 (33%)	3 (27%)	4 (31%)	11 (26%)	1 (25%)
Ibrutinib	0	0	0	0	0	0
Ibrutinib + venetoclax	43 (33%)	4 (12%)	1 (9%)	4 (31%)	8 (19%)	2 (50%)
Venetoclax + obinutuzumab	16 (12%)	11 (33%)	1 (9%)	2 (15%)	8 (19%)	1 (25%)
*p*‐value for association	*p* < 0.001	*p* = 0.002	*p* = 0.013	NS	*p* = 0.008	NS

Abbreviation: NS, not‐significant.

Concomitant medications additionally influenced treatment choice (Table 3). When comparing treatment allocation between patients receiving and not receiving specific concomitant therapies, no significant differences were observed for antiplatelet use (*n* = 50; *p* = 0.0572), although acalabrutinib was the most frequently prescribed regimen in patients on antiplatelet drugs (32%). Conversely, a significant association was observed between direct oral anticoagulant (DOAC) use and treatment choice (*p* = 0.001), with zanubrutinib being more frequently used in DOAC‐treated patients (43%). Patients receiving steroid or other immunosuppressive therapy were most frequently treated with VO (46%) or continuous BTKi (45%). With the caveat of limited sample size (*n* = 11), treatment allocation was significantly impacted in patients receiving versus not receiving these agents (*p* = 0.044).

**TABLE 3:** Allocation of different treatments based on concomitant therapies at baseline.

	Aspirin or other antiplatelet drugs (*n* = 50)	DOAC (*n* = 23)	Steroids or other immuno‐suppressive therapies (*n* = 11)	Vitamin k antagonists (*n* = 3)	CYP3A4 inhibitors (*n* = 3)
Acalabrutinib	16 (32%)	5 (22%)	3 (27%)	0	0
Zanubrutinib	9 (18%)	10 (43%)	2 (18%)	0	0
Ibrutinib	0	0	0	0	0
Ibrutinib + venetoclax	13 (26%)	2 (9%)	1 (9%)	0	0
Venetoclax + obinutuzumab	12 (24%)	6 (26%)	5 (46%)	3 (100%)	3 (100%)
*p*‐value for association	NS (*p* = 0.057)	*p* = 0.001	*p* = 0.044	NS	NS

Abbreviations: DOAC, direct oral anticoagulant; NS, not‐significant.

### 3.5 Socio‐Demographic Characteristics

Data on social support and occupational status were collected to evaluate a potential association with treatment allocation (Table 1).

Work status differed significantly among the treatment groups (*p* < 0.001). Active workers (*n* = 103) were predominantly treated with IV (66%), whereas retired or unemployed patients (*n* = 182) were more frequently managed with continuous BTKi (58%).

In contrast, caregiver availability did not differ significantly across treatment groups.

### 3.6 Physician‐Rated Determinants of Treatment Selection

**FIGURE 3:**
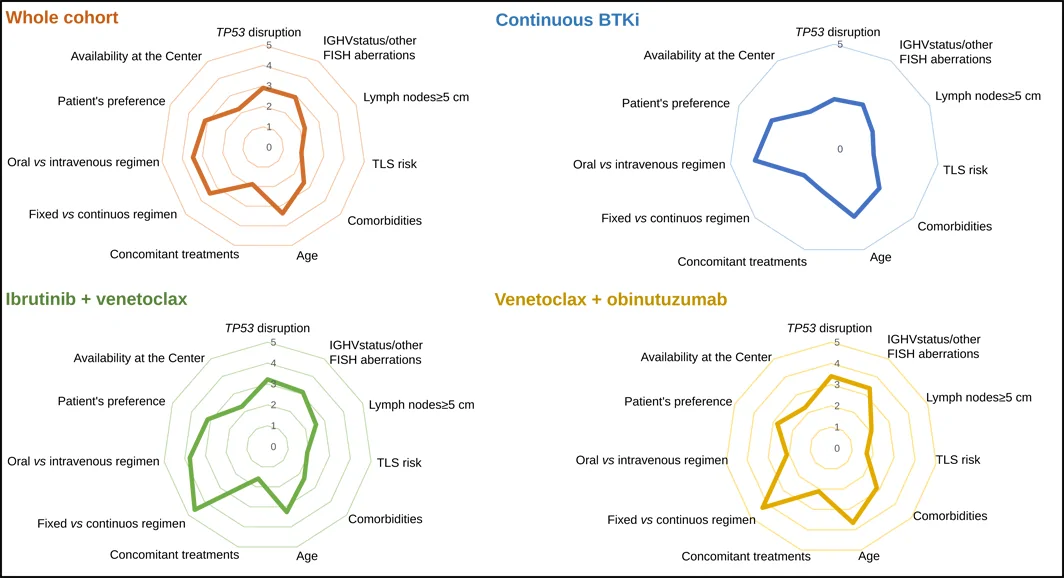
Impact of different parameters on the choice of first‐line treatment, scored according to treating physicians. The spider charts display the mean *relevance score* (1–5) assigned to each decision‐making parameter across treatment groups.

**FIGURE 4:**
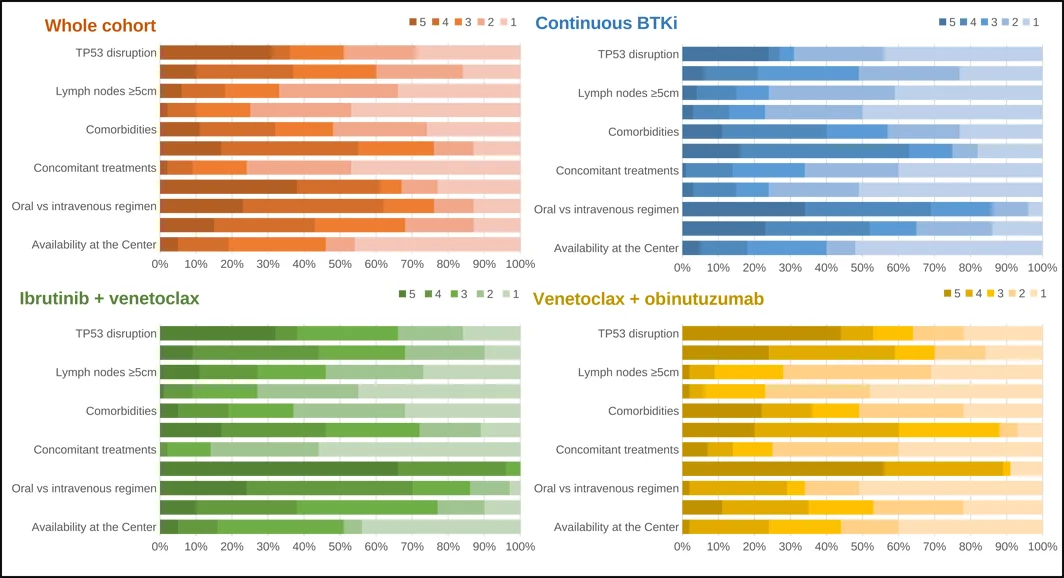
Distribution of physicians' *relevance scores* for treatment decision parameters. The figure displays the percentage of physicians assigning scores from 1 to 5 for each evaluated decision‐making parameter, stratified by treatment group.

Finally, we assessed the physician perceived importance of different clinical, biological and patient‐related parameters in the treatment decision for each patient of the cohort. Figure 3 depicts the impact of each analyzed variable on treatment selection, based on the 1 to 5 *relevance score*. Figure 4 reports the full distribution of the *relevance scores*. Statistical comparisons are shown in Supporting Information S1: Table S1.

Considering the whole cohort, patient's age, route and timing of administration, and patient's preference emerged as the main determinants of therapeutic decision‐making (mean *relevance score*: 3.37, 3.47, 3.45 and 3.13, respectively), while TLS risk and concomitant treatments showed minimal influence (mean *relevance score*: 1.89 and 1.88, respectively).

When treatment groups were analyzed separately, age, oral administration and patient preference resulted the most relevant determinants in the continuous BTKi cohort (mean *relevance score*: 3.36, 3.82 and 3.28, respectively). Regarding venetoclax‐based regimens, the presence/absence of high‐risk factors (*TP53* disruption and IGHV status/other FISH aberrations), age and the fixed‐duration schedule resulted key determinants in treatment choice (mean *relevance score*: 3.22, 3.11, 3.26 and 4.61 for IV; 3.40, 3.36, 3.66 and 4.28 for VO, respectively). In the IV cohort, oral administration and patient preference played an additional role (mean *relevance score*: 3.76 and 3.15, respectively).

When comparing the treatment groups, the importance in treatment decision attributed to *TP53* disruption and IGHV status/other FISH aberrations was significantly lower in the BTKi cohort versus both IV (*p* < 0.001) and VO (*p* < 0.001), whereas no significant difference was observed between IV and VO.

The perceived impact of age in treatment choice was similar across regimens (mean *relevance score*: 3.36 for BTKi, 3.26 for IV and 3.66 for VO). Comorbidities and concomitant treatments received significantly higher *relevance scores* when BTKi was the chosen treatment, compared with IV (*p* = 0.010 and *p* = 0.003, respectively), while no differences were observed compared with VO (mean *relevance score*: 2.85 and 2.08 for BTKi; 2.32 and 1.59 for IV; 2.85 and 2.11 for VO, respectively).

The importance of lymph node size in treatment choice was generally modest, except for the IV group, where it was considered significantly (*p* = 0.003) more relevant compared with VO (mean *relevance score*: 2.01 for BTKi, 2.55 for IV and 2.09 for VO). The impact of TLS risk was consistently rated low across all three treatment groups (mean *relevance score*: 1.90 for BTKi, 1.92 for IV and 1.69 for VO).

Among logistical factors, the importance of treatment schedule differed significantly between the treatment groups: *relevance scores* for route of administration were significantly lower in the VO group compared with both IV and BTKi (mean *relevance score*: 3.82 for BTKi, 3.76 for IV and 2.11 for VO; *p* < 0.001), while *relevance scores* for duration of the therapy were significantly lower in the BTKi group compared with both IV and VO (mean *relevance score*: 1.91 for BTKi, 4.61 for IV and 4.28 for VO; *p* < 0.001). Although overall considered a low‐impact factor, the availability of the regimen at the Center obtained a significantly lower *relevance score* for BTKi compared to VO (*p* = 0.040), with no significant differences compared with IV (mean *relevance score*: 2.12 for BTKi, 2.28 for IV and 2.28 for VO).

Finally, patient's preference was significantly rated as more relevant in the choice of BTKi compared to VO (*p* = 0.040), with no significant differences with the IV cohort (mean *relevance score*: 3.28 for BTKi, 3.15 for IV and 2.79 for VO).

Within the group of patients treated with continuous BTKi, a dedicated comparison between second‐generation compounds was performed (Supporting Information S1: Table S2 and Figure S1). The relevance of comorbidities was rated higher for patients who started acalabrutinib compared to zanubrutinib (mean *relevance score*: 3.05 and 2.68, respectively). Conversely, *TP53* disruption (mean *relevance score*: 2.82 and 1.98, respectively) and IGHV status/other FISH aberrations (mean *relevance score*: 2.79 and 2.25, respectively) emerged as more important in the choice of zanubrutinib compared to acalabrutinib. Nonetheless, the only comparison reaching statistical significance was that of IGHV status/other FISH aberrations (*p* = 0.010).

## 4 Discussion

The present multicenter real‐world study provides a contemporary insight into how first‐line treatment decisions are implemented in routine CLL practice in Italy. In a therapeutic landscape characterized by multiple highly effective chemoimmunotherapy‐free options, treatment allocation is not purely based on efficacy parameters, but appears to reflect a nuanced, regimen‐specific integration of biological risk, comorbidity burden, logistical constraints and patient‐centered considerations, rather than reliance on a single dominant determinant.

Biological risk stratification emerged as a key determinant of therapeutic selection. For *TP53*‐aberrant CLL, current evidence suggests that BTKi improve outcomes more effectively than venetoclax‐based regimens [4, 5, 13, 14, 15]. Accordingly, and in line with European guideline recommendations [1], in our cohort patients harboring *TP53* disruption were preferentially treated with continuous BTKi, particularly zanubrutinib. Although direct comparative data among covalent BTKi are limited, results from the SEQUOIA study demonstrated durable responses in patients with *TP53* disrupted disease [16], partly explaining this preferential selection. IGHV mutational status also retains prognostic significance, with unmutated IGHV being associated with inferior outcomes with fixed‐duration venetoclax‐based combinations [5, 15]. In our cohort, VO was predominantly prescribed in patients with mutated IGHV status, mirroring real‐world evidences [17, 18] and 2024 ESMO recommendations [1]. IGHV status appeared less discriminatory for IV allocation, consistently with other real‐world series [19, 20]. Preliminary data from the CLL17 trial further contextualize these findings, showing inferior outcomes with fixed‐duration regimes, particularly VO, among patients with *TP53* aberration [21]. Accordingly, in our study, the *relevance score* analysis identified *TP53* status as a major driver of continuous BTKi selection and avoidance of fixed‐duration strategies, whereas IGHV status/other FISH aberrations primarily influenced the choice of VO.

As expected, age and comorbidity burden emerged as additional relevant determinants of treatment allocation. Data from the GLOW study raised safety concerns regarding the use of the IV regimen in patients with a high comorbidity burden [22]. Consistently, in our cohort the elderly population was preferentially treated with continuous BTKi, and these regimens were also favored in patients with higher CIRS scores and comorbidities. Specifically, the frequent use of BTKi in patients with cardiovascular disorders aligns with observations from real‐world studies [23, 24], supporting the notion that traditional cardiovascular risk factors are no longer considered absolute contraindications to BTKi therapy. The CLL‐frail trial further supports this strategy, demonstrating maintained efficacy and manageable safety of acalabrutinib in frail patients or in those aged ≥ 80 years [25]. The evaluation of concomitant therapies reinforced this interpretation. The use of DOAC or antiplatelet drugs did not preclude BTKi prescription, suggesting growing confidence in the safety profile of second‐generation BTKi. These patterns are further supported by the *relevance score* analysis: age consistently ranked among the most influential determinants of treatment selection across the cohort, while comorbidity burden showed a particularly strong impact on guiding the choice of continuous BTKi. Although concomitant therapies received overall low *relevance score*, their impact was significantly greater in the BTKi group, reflecting the higher comorbidity burden of these patients. Of note, we acknowledge that in our study patients' fitness was assessed using the CIRS score, that was primarily developed in the context of chemotherapy‐based trials.

Regarding logistical factors, the fixed versus continuous treatment schedule and the route of administration were among the highest‐rated factors for treatment decision, particularly for fixed‐duration regimens. In routine clinical practice, continuous BTKi are typically associated with a lower burden of hospital visits compared to infusion‐based regimens. Conversely, pharmacoeconomic data indicate that healthcare costs decline substantially after completion of fixed‐duration VO, whereas no comparable reduction is observed with treat‐to‐progression BTKi [26]. These findings highlight the dynamic trade‐off between early monitoring intensity and long‐term economic sustainability.

Overall, caregiver support was available for the vast majority of patients, possibly attenuating the results regarding the impact of social support on treatment allocation. Also, occupational status was associated with treatment choice, but likely reflected age differences across groups.

Importantly, patient‐centered variables ranked among the most influential determinants of treatment choice in our whole cohort. Our findings align with discrete choice experiments and physician‐focused analyses demonstrating that treatment duration, route of administration, safety profile, quality of life and patient preference substantially influence therapeutic dicisions [27, 28, 29, 30, 31]. However, unlike discrete choice experiments—based on hypothetical scenarios—and prior physician‐focused studies that evaluated a limited number of treatment options, our analysis captures real‐world decision‐making across the full spectrum of currently available frontline therapies.

Collectively, our data contributes to redefining first‐line CLL decision‐making as a hierarchical and multidimensional process. High‐risk molecular features—particularly *TP53* disruption—appear to function as primary filters. Among patients without adverse molecular characteristics, treatment allocation becomes shaped by clinical complexity, feasibility considerations and patient values. This transition reflects a pragmatic adaptation of trial‐derived evidence to the heterogeneity of real‐world populations.

It should be highlighted that, in the present study, treatment allocation was not randomized and reflected physician discretion: the *relevance score*, although prospectively collected, remains subjective and may vary across clinicians. However, the study involved a substantial number of treating physicians across Centers with extensive experience in CLL management, representing a strength of the analysis. Furthermore, the study was conducted within a single national healthcare system, and treatment availability was restricted to regimens reimbursed in Italy, thereby increasing the homogeneity of the sample, but also potentially limiting external validity. Also, subgroup analyses were limited by small sample size, and the absence of multivariable modeling limits the ability to disentangle independent predictors of treatment allocation. Finally, clinical outcomes were not described, but future follow‐up might provide this data. Nonetheless, this study represents one of the first structured, multicenter real‐world efforts to quantitatively assess how different determinants are weighted in frontline CLL treatment selection, offering a data‐driven framework to interpret clinical choices beyond descriptive patterns.

In conclusion, our findings indicate that contemporary CLL management is increasingly characterized by therapy‐specific prioritization rather than uniform algorithms. The integration of real‐world decision‐making data with outcome‐based evidence may represent a crucial step toward refining personalized treatment strategies and informing future guidelines for CLL.

## Author Contributions

F.P. and C.V. designed and coordinated the research, provided patient care, collected data, analyzed and interpreted the data, and wrote the manuscript. M.A., R.P., S.P., M.N., M.D., I.P., E.F., A.B., R.M., A.F., M.S., E.B., M.B., M.F., A.G., M.C., G.G., M.M., C.S, R.M., A.M.F., R.F., J.O., F.R.M. and B.B. provided patient care, collected data, contributed to data interpretation and discussed results. All the authors contributed to manuscript revision and approved the submitted version.

## Funding

The authors have nothing to report.

## Ethics Statement

The study was approved by the Institutional Review Board (IRB) “Comitato Etico Interaziendale A.O.U. Città della Salute e della Scienza di Torino” (protocol code UniTO‐CLL11; practice number CET00116/2025) and by the Institutional Review Boards/Ethics Committees of all participating centers. The study was conducted in accordance with the Declaration of Helsinki.

## Consent

Written informed consent was obtained from all patients prior to study enrollment.

## Conflicts of Interest

Francesca Perutelli speaker’s honoraria: AstraZeneca, Lilly, Takeda, Roche; travel support: Johnson & Johnson. Riccardo Moia advisory board and speaker’s honoraria: Johnson & Johnson, Abbvie, AstraZeneca, BeOne. Roberta Murru: advisory boards: AbbVie, AstraZeneca, Beone, and Johnson & Johnson; travel grants and speaking honoraria: AbbVie, AstraZeneca, Beone, Eli Lilly and Johnson & Johnson. Marta Coscia research support: Abbvie, GSK; Johnson & Johnson; advisory board/speaker’s bureau: Abbvie, AstraZeneca, Behring, BeOne, Bristol Myers Squibb, GSK, Johnson & Johnson, Lilly; travel support: Johnson & Johnson, BeOne, AstraZeneca, AbbVie. Gianluca Gaidano research support: Gilead; advisory board/speaker’s bureau: Abbvie, AstraZeneca, BeOne, Hikma, Incyte, Johnson & Johnson, Lilly. Jacopo Olivieri advisory board: Abbvie, BMS, Roche; speakers’ bureau: Abbvie, Astrazeneca, BeOne, BMS, Incyte, Takeda, Kite, Eli Lilly, Janssen, Novartis, Roche, SOBI. Francesca Romana Mauro advisory board: Abbvie, AstraZenca; speaker’s bureau: Abbvie, AstraZenca, BeOne. Travel support: Abbvie. Candida Vitale consultancy fees/advisory board: Johnson & Johnson, Abbvie, BeOne; travel support: Johnson & Johnson, Abbvie, BeOne, Astra Zeneca, Lilly; speakers’ bureau: Lilly. The remaining authors have no conflict of interest to declare.

## Permission to Reproduce Material From Other Sources

The authors have nothing to report.

## Supporting information


Supporting Information S1

## Data Availability

De‐identified individual participant data underlying the results reported in this article will be made available upon reasonable request following publication, subject to approval by the investigators and in compliance with applicable ethical approvals and data protection regulations. Requests for access to the data, including original data, should be addressed to the Corresponding Author.
