# Supplementary material for: Real‐World Determinants of Treatment Selection in Frontline Chronic Lymphocytic Leukemia: The FIRST‐CLL Study
Source: Hematol Oncol. 2026 Sep 16;44(5):e70246. doi: 10.1002/hon.70246 (PMC13580107; doi:10.1002/hon.70246)
Supplement: Supplementary file 1 [file HON-44-e70246-s001.docx]

**Supplemental data**

**Real-world determinants of treatment selection in frontline chronic lymphocytic leukemia: the FIRST-CLL study**

**Supplemental Table S1. Mean *relevance scores* for the analyzed parameters potentially impacting the choice of treatment.**

|  | **Whole cohort**  **(n=285)** | **BTKi**  **(n=116)** | **IV**  **(n=114)** | **VO**  **(n=55)** |
| --- | --- | --- | --- | --- |
| *TP53* disruption | 2.91 | **2.38** | **3.22** | **3.40*** |
| IGHV status/other FISH aberrations | 2.91 | **2.52** | **3.11** | **3.36^** |
| Lymph nodes ≥5 cm | 2.24 | 2.01 | **2.55** | **2.09°** |
| TLS risk | 1.89 | 1.90 | 1.92 | 1.69 |
| Comorbidities | 2.64 | **2.85** | **2.32^#^** | 2.85 |
| Patient's age | 3.37 | 3.36 | 3.26 | 3.66 |
| Concomitant treatments | 1.88 | **2.08** | **1.59^@^** | 2.11 |
| Fixed duration vs continuous therapy | 3.45 | **1.91** | **4.61** | **4.28*** |
| Oral vs intravenous therapy | 3.47 | **3.82** | **3.76** | **2.11^+^** |
| Patient's preference | 3.13 | 3.28 | 3.15 | 2.79 |
| Availability of the regimen at the Center | 2.22 | **2.12** | 2.28 | **2.28^£^** |

Abbreviations: FISH, fluorescence in situ hybridization; IGHV, immunoglobulin heavy chain variable region; TLS, tumor lysis syndrome.

*BTKi vs IV, p<0.001 when comparing; BTKi vs VO, p<0.001.

^BTKi vs IV, p<0.001; BTKi vs VO, p<0.001.

° p=0.003 for all comparisons.

^#^ p=0.010 for all comparisons.

**^@^** p=0.003 for all comparisons.

**^+^** VO vs BTKi, p<0.001; VO vs IV, p<0.001.

^£^ p=0.040 for all comparisons.

**Supplemental Table S2.** Mean *relevance score*s of the parameters impacting the choice of treatment with acalabrutinib and zanubrutinib.

|  | **Acalabrutinib**  **(n=59)** | **Zanubrutinib**  **(n=54)** |
| --- | --- | --- |
| *TP53* disruption | 1.98 | 2.82 |
| IGHV status/other FISH aberrations | **2.25** | **2.79*** |
| Lymph nodes ≥5 cm | 2.06 | 1.93 |
| TLS risk | 1.93 | 1.84 |
| Comorbidities | 3.05 | 2.68 |
| Patient's age | 3.46 | 3.30 |
| Concomitant treatments | 2.08 | 2.09 |
| Fixed duration vs continuous therapy | 1.71 | 2.13 |
| Oral vs intravenous therapy | 3.88 | 3.77 |
| Patient's preference | 3.26 | 3.21 |
| Availability of the regimen at the Center | 2.10 | 2.16 |

Abbreviations: FISH, fluorescence in situ hybridization; IGHV, immunoglobulin heavy chain variable region; TLS, tumor lysis syndrome.

*p=0.010

**Supplemental Figure 1.** Distribution of physicians’ *relevance scores* (1–5) and impact of each decisional parameters in the choice of acalabrutinib or zanubrutinib treatment.
